# *CBC*ovid19EC: A dataset complete blood count and PCR test for COVID-19 detection in Ecuadorian population

**DOI:** 10.1016/j.dib.2023.109016

**Published:** 2023-03-01

**Authors:** R. Ordoñez-Avila, J. Parraga-Alava, J. Meza Hormaza, L. Vaca-Cárdenas, E. Portmann, L. Terán, M. Dorn

**Affiliations:** a Facultad de Ciencias Informáticas, Universidad Técnica de Manabí, Portoviejo, Manabí, Ecuador; b University of Fribourg, Fribourg, Switzerland; c Department of Theoretical Informatics, Institute of Informatics, Federal University of Rio Grande do Sul, Porto Alegre, RS, Brazil; d Center of Biotechnology, Federal University of Rio Grande do Sul, RS, Brazil; e National Institute of Science and Technology, Forensic Science, Porto Alegre, RS, Brazil; f Lucerne University of Applied Sciences and Arts, Switzerland

**Keywords:** SARS-Cov-2, Hematological data, Machine learning, Ecuador

## Abstract

In this work, we present the complete blood count data and PCR test results of a population of Ecuadorians from different provinces, primarily residing in the Andean region, especially in Quito. PCR was the standard test to detect Covid-19 during the pandemic since 2020. The data were obtained between March 1st and August 12th, 2021. *Segurilab* and *Previne Salud* laboratories performed the tests. The dataset contains about 400 clinical cases. Each patient agreed to participate in the study by sharing the results of their PCR (reverse transcription polymerase chain reaction) tests and CBC (complete blood count). CBC test measured several components and features of the blood, including red blood cells, white blood cells, hemoglobin, hematocrit, and platelets. The shared data are intended to provide researchers with input to analyze various events associated with the diagnosis of Covid-19 linked to potential diseases identified in the components measured in the CBC test. These data are helpful for pattern analysis of blood components in modeling prediction and clustering problems. The components measured in the complete blood count and CRP together can be helpful for the analysis of different medical conditions using machine learning algorithms.

**Specifications Table**

**Table:** 

Subject	Machine Learning
Specific subject area	COVID-19 Diagnostics
Type of data	Table
How data were acquired	The PCR and CBC test samples were taken from men and women of different ages in the city of Quito through the *Segurilab* and *Previne Salud* laboratories. The PCR test has been used for early and reliable diagnosis possible by swabbing the nasopharynx [1]. The type of CBC test is hematology, which studies the blood and its disorders. For CBC, manual microscopy counts are still required when a sample is flagged by the hematology analyzer [2]. The patients agreed to participate in the study utilizing an agreement signed by them. The tests were performed without any order or priority during the year 2021.
Data format	Raw and Processed CSV format.
Description of data collection	Hemogram data were captured before, during, or after PCR sampling; a necessary condition was to capture data for both genders and different ages in children, adolescents, adults, and older adults. Both tests were performed in Quito, Ecuador, between March 1st and August 12th, 2021. Data obtained by PCR test were performed on nasopharyngeal specimens for the diagnosis of SARS-Cov-2 infection. In addition, an identifier feature is included for each case, the positive or negative result for COVID-19, available in the PCR characteristic, the date of the CBC test and of the PCR test sample collection, sex, age and 20 characteristics referred to the blood components measured in the blood count test.
Data source location	City/Town/Region: Quito – Ecuador. Latitude and longitude: -0.22985, -78.52495.
Data accessibility	Repository name: CBCovid19EC Data identification number: DOI: 10.17632/7bmfgkkm3z.3 Direct URL to data: https://data.mendeley.com/datasets/7bmfgkkm3z/3

## Value of the Data

• The data provided favor the identification of medical conditions by analyzing patterns in the values of the CBC test associated with the results of the infection caused by SARS-Cov-2.
• The shared blood component values and COVID-19 results can be used for research separately or in children up to older adults of both genders.
• These data are suitable for training and benchmarking machine learning algorithms, focusing on prediction, and clustering techniques.
• The CBC test data can serve as input for other investigations that attempt to discover the comparative ethnic characterization of Latin American populations using data from an Ecuadorian population as a reference.
• The data can motivate the characterization of a patient's condition, through measurements of blood components, in association with age and gender.
• Governments can use the CBC as a tool for COVID-19 diagnosis in scenarios of insufficient tests. Hemogram results from asymptomatic and symptomatic individuals could be used as an alternative to detect infections reducing the application of costly tests such as the PCR test and thereby saving economic resources.

## 1 Objective

The dataset was created to allow researchers to analyze the prediction and clustering of the CBC test values, together with the PCR test results, within the machine learning framework.

## 2 Description of Data

The data set presents information from PCR as a commonly used test for diagnosing SARS-Cov-2 infection by obtaining a positive or negative result for Covid-19. The CBCovid19EC dataset includes values from the CBC test, describing blood components measured in men and women from 7 to 82 years of age. The blood biometry tests were performed in private laboratories in Quito, with samples taken from patients from different provinces of Ecuador. The patients agreed to share their data by participating in this study. In total, 377 records included data on age, gender, and date of sample collection for PCR and CBC examination. In the processed data, an identifier feature is included for each case, the positive or negative result for COVID-19, available in the PCR characteristic, the date of the CBC test and the PCR test sample collection, sex, age, and twenty characteristics referred to the blood components measured in the blood count test. The raw data includes provincial characteristics such as the patient's place of origin, the laboratory, and the test type. These data were excluded from the processed data.

**Fig. 1:**
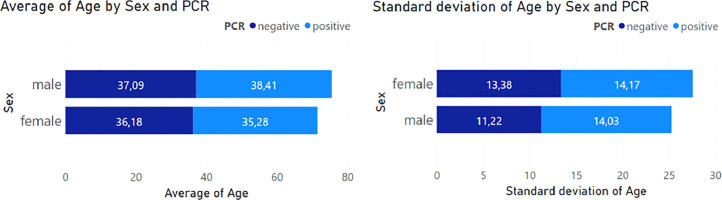
The average and standard deviation of age and sex of patients with positive and negative PCR samples.

Table 1 shows the positive and negative results of the diagnosis of SARS-Cov-2 infection, adding the data's mean and variance, the patients' age range, and the number of samples obtained for each diagnosis. Fig. 1, on the left, is the summary reflecting some negative samples outnumbering positive ones by a smaller amount.

**Table 1:** PCR results by sex, including age data: mean, standard variation, range, and sample count.

PCR Positive			PCR Negative		
Male	Female	Total	Male	Female	Total
38.41 ± 14.12	35.28 ± 14.32	37.17 ± 12.23	37.09 ± 11.26	36.18 ± 13.46	36.76 ± 12.08
12 ∼ 81	7 ∼ 78	7 ∼ 81	13 ∼ 82	7 ∼ 72	7 ∼ 82
76	50	126	160	91	251

In Fig. 1 we can observe that Positive PCR samples are higher in men with an average age of 38 years, while women with an average age of 35 have positive PCR samples. On average, negative PCR samples occur between 37 and 36 years of age in men and women.

**Fig. 2:**
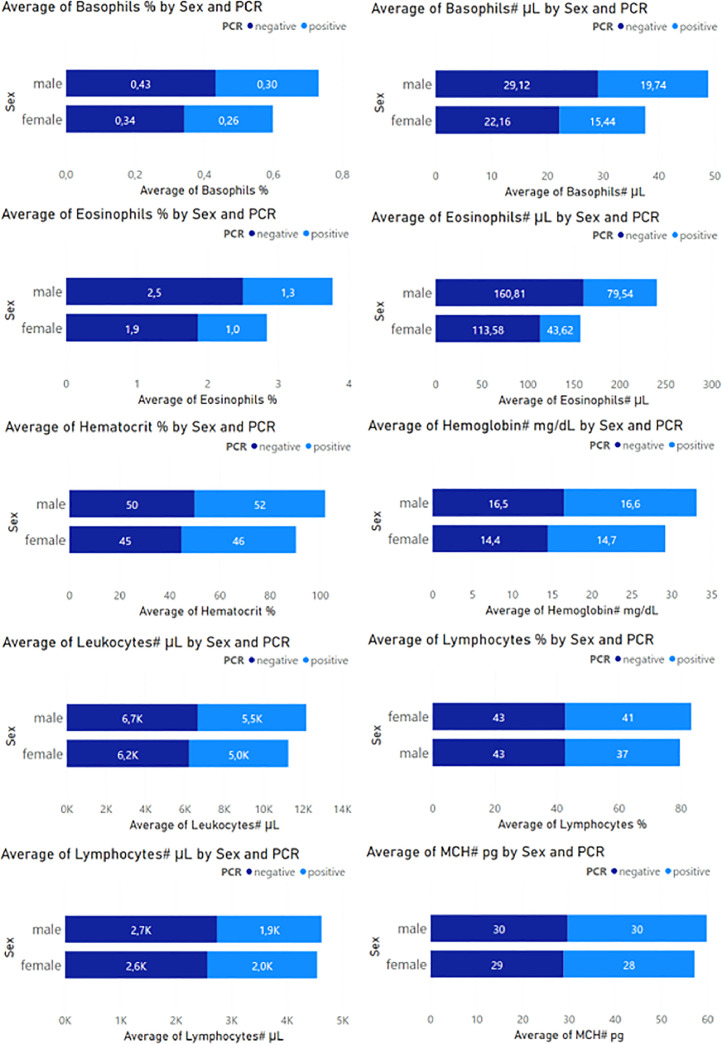
The first set of blood component values of the CBC test.

**Fig. 3:**
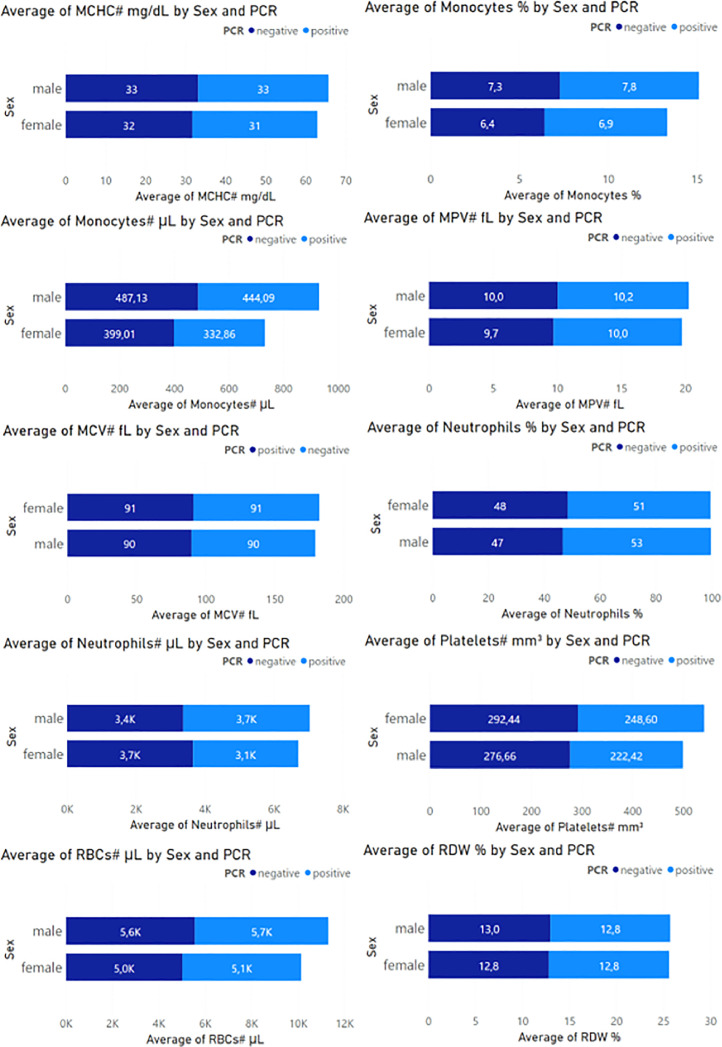
Second set of blood component values of the CBC test.

Figs. 2 and 3 present the averages of the blood component values obtained in the CBC test, separated by two groups of visualizations. A set of Figs. describing the summary data, combining the sex data and the PCR test results, for each of the blood components. The statistic used to present the summary data is the arithmetic mean, which helps to identify the distribution of the data, knowing that most of the measurements correspond to negative PCR values. Some blood components are presented with values in percentages and others in their units of measurement.

In Fig. 2 we can observe that Basophils are present in more significant quantities in negative PCR samples, with males at 0.43% and females at 0.34% on average. In negative PCR samples, eosinophils have a higher average percentage, present in 2.5% of males and 1.9% females. Hematocrit in greater quantity in positive PCR samples, with an average of 52% in men and 46% in women. Leukocytes were in higher proportion in negative PCR samples, averaging 6700 µL in males and 6200 µL in females. Lymphocytes are present in greater quantity in PCR-negative samples, on average 43% in males and females. MCH, on average, is higher in PCR-negative samples, with 30% in males and 29% in females.

In Fig. 3 we can observe that MCHC, on average, is higher in negative PCR samples, 33 mg/dL in men and 32 mg/dL in women. Monocytes, on the contrary, are higher in positive PCR samples, on average 7.8% in men and 6.9% in women. MPV is also higher in PCR-positive samples, with an average of 10.2 fL in men and 10.0 fL in women. MCV presented equal amounts in positive and negative PCR samples in both males and females, 90 fL and 91 fL, respectively. Neutrophils present in a more significant proportion in positive PCR samples, on average 51% in females and 53% in males. Platelets in greater quantity in negative PCR samples, an average of 292 mm3 in women and 277 mm3 in men. RBCs in greater quantity in positive PCR samples, 5700 uL in men and 5100 uL in women. RDW presents relatively similar proportions in positive and negative PCR samples.

## 3 Experimental Design, Materials and Methods

The *CBCovid19EC* dataset shares information from PCR (*reverse transcription polymerase chain reaction*) and CBC (complete blood count) tests. The complete blood cell count (CBC) is one of the most frequently ordered laboratory tests [3]. The PCR test has been used for early and reliable diagnosis possible by swabbing the nasopharynx. Using a blood sample, the complete blood count provides essential information on blood cells, especially red blood cells, white blood cells, and platelets [4]. Both tests were performed in Quito, Ecuador, between March 1st, 2021, and August 12th, 2021.

The tests were performed by the laboratories *Segurilab*^1^ (213 rows) and *Previene Salud*^2^ (164 rows). The dataset includes 377 cases of COVID-19 diagnosis, presented in a dichotomous variable with negative and positive values. In addition, the age and gender of the patient and 20 features of hemogram information are shared.

Table 2 describes the 26 features of the dataset and the data type. Most of these features are of numerical type, especially one categorical data relating to the PCR test result.

**Table 2:** Description of variables available in CBCovid19EC dataset**.**

Number	Variable	Description	TYPE
1	id	Identifier column	Identifier
2	sex	Sex of patient	Character
3	age	Patient's age	Numeric
4	pcr_date	PCR sample date	Date
5	exam_bh_date	Date of hemogram examination	Date
6	leukocytes	Leukocytes # (µL)	Numeric
7	neutrophilsP	Neutrophils (%)	Numeric
8	lymphocytesP	Lymphocytes (%)	Numeric
9	monocytesP	Monocytes (%)	Numeric
10	eosinophilsP	Eosinophils (%)	Numeric
11	basophilsP	Basophils (%)	Numeric
12	neutrophils	Neutrophils # (µL)	Numeric
13	lymphocytes	Lymphocytes # (µL)	Numeric
14	monocytes	Monocytes # (µL)	Numeric
15	eosinophils	Eosinophils # (µL)	Numeric
16	basophils	Basophils # (µL)	Numeric
17	redbloodcells	RBCs is red blood cells # (µL)	Numeric
18	mcv	MCV is the mean corpuscular volume (fL)	Numeric
19	mch	MCH is the mean corpuscular hemoglobin (pg)	Numeric
20	mchc	MCHC is the Mean corpuscular hemoglobin concentration (mg/dl)	Numeric
21	rdwP	RDW is Red Blood Cell Distribution Width (%)	Numeric
22	hemoglobin	Hemoglobin (mg/dl)	Numeric
23	hematocritP	Hematocrit (%)	Numeric
24	platelets	Platelets (mm³)	Numeric
25	mpv	MPV is the mean platelet volume	Numeric
26	pcr	Positive or negative results for Covid-19	Categorical

There were 251 cases of negative results for Covid-19, in contrast to less than 40% of samples, represented by 126 positive cases. In addition, 141 women and 236 men participated in this study, with an average age of 37 years in both cases. To identify the average values of the blood components measured in the CBC test, these were grouped with the characteristics of sex and PCR.

In Fig. 2, Basophil values averaged 0.43% in males and 0.34% in females for PCR-positive cases, while the values for PCR-negative cases were 0.30 in males and 0.26 in females. The number of basophils exceeds in negative cases with a value above 29 µL in males and 22 µL in females compared to positive cases. Eosinophils present average values of 2.5% in men and 1.9% in women for negative cases, exceeding the positive PCR results with values of 160.81 µL in men and 113.58 in women. Leukocytes, Lymphocytes, Monocytes, Neutrophils, Plaletets, and RBCs, also present variations in their measurements, maintaining higher negative results. These values are presented in Fig. 3.

In Fig. 2, Hematocrit, on the other hand, presents similar measurements for both PCR results, with 50% in men and 45% in women for negative cases, in balance with 52% in men and 46% in women for positive cases. Similarly, hemoglobin values were quite similar, with measurements of 16.5 mg/dL in men and 14.4 mg/dL in women for negative cases, as well as 16.6 mg/dL in men and 14.7 mg/dL in women for positive cases. Likewise, in Fig. 3, MCH, MCHC, MPV, MCV, and RDW present relative values, and their measurements do not differ significantly.

In summary, there are more samples with negative than positive values. The values of the blood components that present differences in the negative and positive results for Covid-19 are: Basophils, Eosinophils, Leukocytes, Lymphocytes, Monocytes, Neutrophils, Plaletets, and RBCs, while the blood components: Hematocrit, Hemoglobin, MCV, MCH, MCHC, MPV, MCV, and RDW are represented in similar or balanced values for both PCR results.

## Ethics Statement

The review committee approved the study at the Technical University of Manabí with protocol number CBI-UTM-20-11-20-ROA. Every Laboratory informed the study motivation and obtained patients' willingness before practicing the test. In the case of underage participants, informed consent was obtained from parents or legal guardians.

## CRediT authorship contribution statement

**R. Ordoñez-Avila:** Data curation, Writing – original draft, Visualization, Validation, Writing – review & editing. **J. Parraga-Alava:** Conceptualization, Methodology, Supervision, Writing – review & editing. **J. Meza Hormaza:** Supervision, Writing – review & editing. **L. Vaca-Cárdenas:** Validation, Writing – review & editing. **E. Portmann:** Validation, Writing – review & editing. **L. Terán:** Validation, Writing – review & editing. **M. Dorn:** Validation, Writing – review & editing.

## Declaration of Competing Interest

The authors declare that they have no known competing financial interests or personal relationships that could have appeared to influence the work reported in this paper.

## Data Availability

CBCovid19EC: A dataset Complete Blood Count and PCR test for COVID-19 detection in Ecuadorian population (Original data) (Mendeley Data).

## Footnotes

1 https://segurilab.ec

2 https://previenesalud.com

## References

[1] Kerimov D., Tamminen P., Viskari H., Lehtimäki L., Aittoniemi J. (Nov. 2021). Sampling site for SARS-CoV-2 RT-PCR-An intrapatient four-site comparison from Tampere, Finland. PLoS One.

[2] Costa L.Da (Mar. 2015). Digital image analysis of blood cells. Clin. Lab. Med..

[3] May J.E., Marques M.B., Reddy V.V.B., Gangaraju R. (Mar. 2019). Three neglected numbers in the CBC: the RDW, MPV, and NRBC count,” *Cleve*. Clin. J. Med..

[4] Feltes B.C. (Mar. 2022). Feature selection reveal peripheral blood parameter's changes between COVID-19 infections patients from Brazil and Ecuador. Infect. Genet. Evol..

